# CD44s and CD44v6 Expression in Head and Neck Epithelia

**DOI:** 10.1371/journal.pone.0003360

**Published:** 2008-10-09

**Authors:** Brigitte Mack, Olivier Gires

**Affiliations:** 1 Department of Otorhinolaryngology, Head and Neck Surgery, Grosshadern Medical Center, Ludwig-Maximilians-University of Munich, Munich, Germany; 2 Clinical Cooperation Group Molecular Oncology, Helmholtz-Zentrum München, German Research Center for Environmental Health, Head and Neck Research Department, Ludwig-Maximilians-University of Munich, Munich, Germany; Karolinska Institutet, Sweden

## Abstract

**Background:**

CD44 splice variants are long-known as being associated with cell transformation. Recently, the standard form of CD44 (CD44s) was shown to be part of the signature of cancer stem cells (CSCs) in colon, breast, and in head and neck squamous cell carcinomas (HNSCC). This is somewhat in contradiction to previous reports on the expression of CD44s in HNSCC. The aim of the present study was to clarify the actual pattern of CD44 expression in head and neck epithelia.

**Methods:**

Expression of CD44s and CD44v6 was analysed by immunohistochemistry with specific antibodies in primary head and neck tissues. Scoring of all specimens followed a two-parameters system, which implemented percentages of positive cells and staining intensities from − to +++ (score = %×intensity; resulting max. score 300). In addition, cell surface expression of CD44s and CD44v6 was assessed in lymphocytes and HNSCC.

**Results:**

In normal epithelia CD44s and CD44v6 were expressed in 60–95% and 50–80% of cells and yielded mean scores with a standard error of a mean (SEM) of 249.5±14.5 and 198±11.13, respectively. In oral leukoplakia and in moderately differentiated carcinomas CD44s and CD44v6 levels were slightly increased (278.9±7.16 and 242±11.7; 291.8±5.88 and 287.3±6.88). Carcinomas *in situ* displayed unchanged levels of both proteins whereas poorly differentiated carcinomas consistently expressed diminished CD44s and CD44v6 levels. Lymphocytes and HNSCC lines strongly expressed CD44s but not CD44v6.

**Conclusion:**

CD44s and CD44v6 expression does not distinguish normal from benign or malignant epithelia of the head and neck. CD44s and CD44v6 were abundantly present in the great majority of cells in head and neck tissues, including carcinomas. Hence, the value of CD44s as a marker for the definition of a small subset of cells (*i.e.* less than 10%) representing head and neck cancer stem cells may need revision.

## Introduction

CD44, the receptor for hyaluronan [1], is encoded by a single gene on chromosome 11p13, but actually represents a polymorphic group of transmembrane glycoproteins owing to extensive alternative splicing and post-translational modifications [2]. The human gene is composed of 19 exons, 10 of which (exons 1–5 and 15–19) are included in the standard form of CD44 termed CD44s. Isoform CD44s is the smallest and most abundant member of this polymorphic and monogenic family of proteins. The remaining exons can be differentially inserted into the mature mRNA via alternative splicing and may in theory give rise to hundreds of protein variants. So far approximately 20 CD44 isoforms were detected in tissues of various origins. In addition to alternative splicing, diversity is added upon varying N- and O-glycosylation sites within the extracellular domain of alternatively spliced products of CD44. In other words, one gene gives rise to a plethora of proteins with potentially discriminative capacities [3].

CD44 was initially identified in the murine lymphoid compartment as a receptor for extracellular matrix (ECM) [4]. Functionally speaking, CD44 is involved in organ integrity through its ability to contact ECM, is signalling active, and serves as a co-receptor for numerous transmembrane proteins such as matrix metalloproteases, members of the ERB family of receptor tyrosine kinases, and the long-known tumour-associated antigen EpCAM (CD326, ESA1) [5]. CD44 was in the focus of molecular oncology in the early 1990s when it was recognized that variants of it, chiefly CD44v6, regulate tumour progression, invasion, and metastasis formation [6]. As has been shown for a number of other receptors, CD44 is dependent on regulated intramembrane proteolysis (RIP) for full activation of its ECM-binding capacity and especially for the induction of signaling properties [7]–[10]. Like the Notch-1 receptor, CD44 is cleaved by ADAM proteases and gamma-secretases to generate an intracellular domain (CD44-ICD), which translocates into the nucleus and fosters transcription of genes from TPA-responsive elements [11].

More recently, the attention paid to CD44s was re-fueled by reports on its differential expression on cancer stem cells (CSCs) versus non-tumourigenic carcinoma cells. In breast [12], colon [13], and HNSCC [14], [15], CD44-positivity was a discriminative characteristic of cancer-initiating cells. In these entities CD44^+^/CD24^−^, CD44^+^/EpCAM^high^, or CD44^+^/Bmi-1^high^ cells but not CD44^−^ cells were able to recapitulate human tumours *in vivo* in immunocompromised mouse recipients. For the specific case of HNSCC, CD44^+^ CSCs were reported to represent a minority population of less than 10% of the tumour cells in human primary carcinomas and did hold stem cell-like properties, *i.e.* self-renewal and pluripotency to some degree [15], [16]. Notedly, the frequency of CSCs in the CD44-positive fraction of HNSCC was one order of magnitude below CSCs from colon or breast carcinomas [16]. Additionally, high-level expression of the transcription factor BMI-1, co-staining with the basal cell marker keratin 5/14, and the lack of involukrin were indicative markers of CSC property in HNSCC [14], [15]. As for the case of the CSC marker CD133 in colon cancer [17], the literature on CD44 expression pattern in HNSCC and its deduced capacity as a CSC marker may appear somewhat controversial. Several research groups described a robust expression of CD44s and/or CD44v6 in head and neck, independently of the malignant state and in a large proportion of tumour cells [18]–[21]. This is clearly in contrast to reports on CD44s as being expressed in less than 10% of HNSCC cells-independently of additional markers used to characterise this minor but highly efficacious tumour cell population.

In the present study, we have attempted to clarify this issue and assessed the expression of CD44s and of tumour-associated splice variant CD44v6 in epithelia of the head and neck area. Specimens collection included normal epithelia, oral leukoplakia, and full-blown primary human carcinomas of varying grading. In the samples studied, we could not recapitulate a differential expression of CD44s that would characterise carcinoma cells versus normal epithelia. CD44s expression in normal and diseased tissue was in every case above 50% of cells. Although differences between normal and transformed tissue were higher than for the case of CD44s, CD44v6 was not an adequate marker either. Further on, owing to the high incidence of CD44s expression in HNSCC its value as a major CSC marker for this specific entity may need to be thoroughly discussed.

## Results

### CD44 in head and neck epithelia: normal mucosa

**Figure 1:**
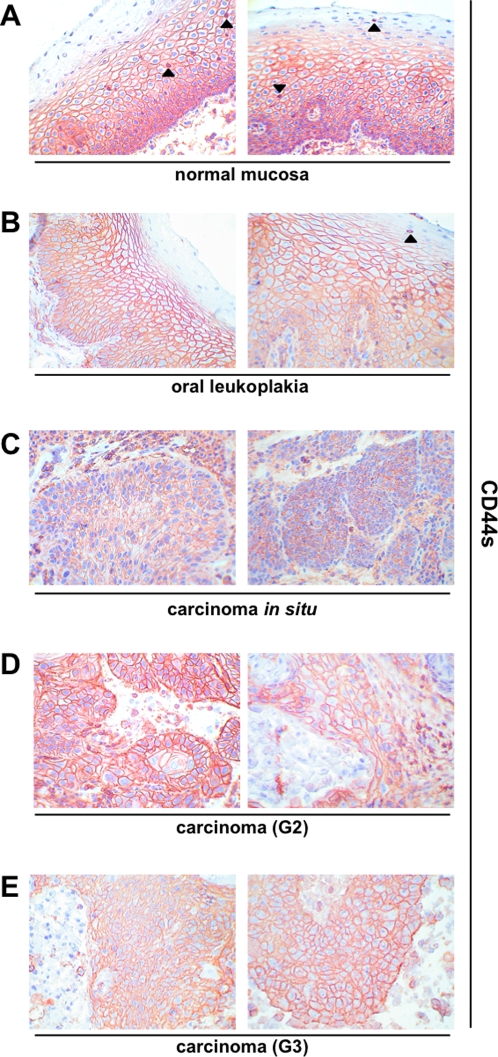
CD44s expression pattern in head and neck epithelia. CD44 standard form was visualised upon immunohistochemistry (reddish brown) in normal epithelium (A), oral leukoplakia (B), carcinomas *in situ* (C), grade 2 carcinomas (D), and grade 3 carcinomas (E). Cell nuclei are stained with hematoxylin (blue). Shown are representative sections. Arrowheads mark infiltrating lymphocytes with high-level CD44s expression.

**Figure 2:**
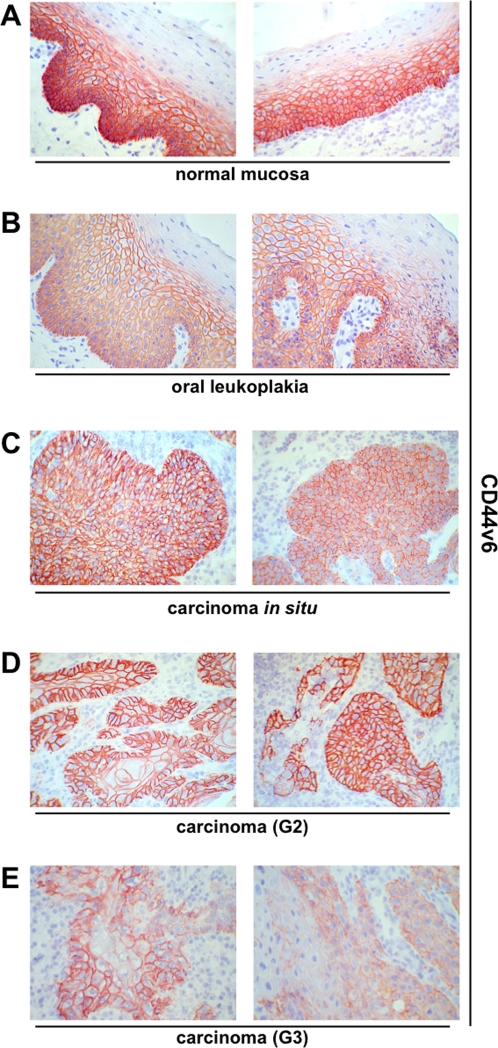
CD44v6 expression pattern in head and neck epithelia. CD44 splice variant 6 was visualised upon immunohistochemistry (reddish brown) in normal epithelium (A), oral leukoplakia (B), carcinomas *in situ* (C), grade 2 carcinomas (D), and grade 3 carcinomas (E). Cell nuclei are stained with hematoxylin (blue). Shown are representative sections.

**Figure 3:**
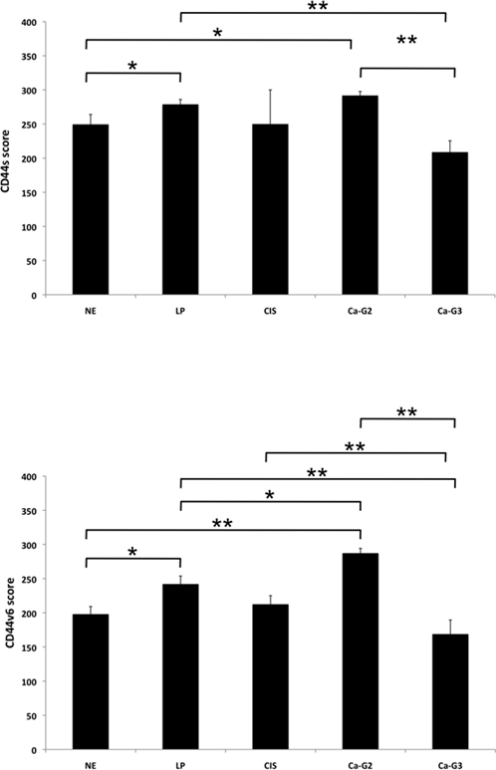
CD44s and CD44v6 expression scores in head and neck epithelia. The expression scores of CD44s (upper) and CD44v6 (lower) implement expression intensity and percentages of positive cells (score = staining intensity×% positive cells; max score 300). Shown are the mean scores with standard deviations. Where indicated, differences across specimen groups were significant (* p<0.05; ** p<0.005). *NE*: normal epithelium; *LP*: leukoplakia; *CIS*: carcinoma *in situ*; *Ca-G2*: grade 2 carcinoma; *Ca-G3*: grade 3 carcinoma.

Before staining, all samples had been confirmed by two pathologists during routine clinical diagnosis, both after instantaneous section and after embedding. Further on, histopathologic examination was conducted after hematoxylin/eosin staining (see Figure S1 for examples of H/E staining). Samples of normal mucosa (n = 10) were stained with CD44s- or CD44v6-specific antibodies. Cell nuclei were visualised with Mayeŕs hematoxylin. For a control consecutive sections were stained with murine pre-immune serum to test for the specificity of the detection system (examples are shown in Figure S2). In average two thirds of the thickness of normal mucosa samples were positive for CD44s and CD44v6 as assessed with a scale in a light microscopy (Figs. 1A and 2A). Additionally antigen expression was recorded as percentage of CD44s- or CD44v6-positive cells and according to expression levels (− to +++). In normal mucosa a strong (+++) and primarily plasma membrane-associated expression of CD44s and CD44v6 was observed in cells of the basal membrane layer and the suprabasal *stratum spinosum* (Figs. 1A and 2A). Cells of the *stratum granulosum* were almost devoid of CD44s and entirely devoid of CD44v6. In opposition to carcinoma samples, a cytoplasmic staining of CD44s and CD44v6 was seen in normal mucosa. This finding may point towards a more efficient shedding of the CD44 ectodomain, although this remains to be shown experimentally. The percentage of CD44s and CD44v6-positive cells within normal epithelia ranged from 60–95% (mean and SEM: 86%±3.5%) and 50%–80% (mean and SEM: 66%±3.7%), respectively (Table 1). Standard error of a mean (SEM) was used in order to include the cohort size into the calculation of deviations, where SEM represents the standard deviation divided by the square root of the sample number. With the purpose to exhibit both parameters, *i.e.* percentages of positive cells and staining intensity at once, we have multiplied both parameters. No staining scored zero while low to strong expression obtained values from 1 to 3. Accordingly, maximal resulting score is 300. The mean scores and SEM for CD44s and CD44v6 in normal mucosa of the head and neck were 249.5±14.5 and 198±11.13 (Fig. 3).

**Table 1:** CD44s and CD44v6 expression in normal epithelia (NE), carcinoma (CA), carcinoma *in situ* (CIS), and leukoplakia (LP).

Tissue	Grading	CD44s (% of cells)	Intensity	Score	CD44v6 (% of cells)	Intensity	Score
**NE**	/	95%	+++	285	50%	+++	150
**NE**	/	60%	++/+++	150	70%	+++	210
**NE**	/	95%	+++	285	80%	+++	240
**NE**	/	80%	++/+++	200	60%	+++	180
**NE**	/	80%	+++	240	70%	+++	210
**NE**	/	95%	+++	285	80%	+++	240
**NE**	/	80%	+++	240	60%	+++	180
**NE**	/	95%	+++	285	80%	+++	240
**NE**	/	95%	+++	285	60%	+++	180
**NE**	/	80%	+++	240	50%	+++	150
**LP**	/	100%	+++	300	80%	+++	240
**LP**	/	90%	+++	270	80%	+++	240
**LP**	/	100%	+++	300	95%	+++	285
**LP**	/	90%	+++	270	80%	+++	240
**LP**	/	80%	+++	240	80%	+++	240
**LP**	/	100%	+++	300	50%	+++	150
**LP**	/	90%	+++	270	50%	+++	150
**LP**	/	100%	+++	300	50%	+++	150
**LP**	/	95%	+++	285	80%	+++	240
**LP**	/	100%	+++	300	90%	+++	270
**LP**	/	90%	+++	270	90%	+++	270
**LP**	/	90%	+++	270	90%	+++	270
**LP**	/	100%	+++	300	90%	+++	270
**LP**	/	50%	+++	150	50%	+++	150
**LP**	/	100%	+++	300	100%	+++	300
**LP**	/	90%	+++	270	90%	+++	270
**LP**	/	90%	+++	270	80%	+++	240
**LP**	/	100%	+++	300	100%	+++	300
**LP**	/	100%	+++	300	100%	+++	300
**LP**	/	100%	+++	300	100%	+++	300
**LP**	/	90%	+++	270	50%	+++	150
**LP**	/	100%	+++	300	100%	+++	300
**CIS**	/	100%	++	200	100%	++	200
**CIS**	/	100%	+++	300	90%	++/+++	225
**CA**	2	100%	+++	300	100%	+++	300
**CA**	2	100%	+++	300	100%	+++	300
**CA**	2	90%	+++	270	90%	+++	270
**CA**	2	100%	+++	300	100%	+++	300
**CA**	2	100%	+++	300	100%	+++	300
**CA**	2	100%	+++	300	100%	+++	300
**CA**	2	80%	+++	240	80%	+++	240
**CA**	2	100%	+++	300	100%	+++	300
**CA**	2	100%	+++	300	100%	+++	300
**CA**	2	100%	+++	300	100%	+++	300
**CA**	2	100%	+++	300	100%	++/+++	250
**CA**	3	70%	+++	210	60%	−/+++	90
**CA**	3	80%	+/+++	160	90%	++	180
**CA**	3	100%	+++	300	100%	++	200
**CA**	3	80%	++/+++	200	60%	−/+++	90
**CA**	3	70%	++/+++	175	60%	−/++	60
**CA**	3	60%	++/+++	150	100%	++	200
**CA**	3	60%	++	120	70%	+/++	105
**CA**	3	80%	++	160	80%	++	160
**CA**	3	80%	+++	240	60%	++/+++	150
**CA**	3	100%	+++	300	100%	+++	300
**CA**	3	100%	++	200	80%	+/+++	160
**CA**	3	100%	++	200	80%	++/+++	200
**CA**	3	100%	+++	300	100%	+++	300

− no staining; + weak; ++ moderate; +++ strong.

The score incorporates the percentage of cells expressing the given antigen multiplied by the staining intensity (0–3). Grading is according to UICC convention.

### CD44 in head and neck epithelia: oral leukoplakia

Oral leukoplakias are at increased risk to progress to carcinoma *in situ* and full-blown carcinomas or may even already display features of cell transformation [22]. Very much in analogy to normal mucosa, expression of CD44s and CD44v6 in leukoplakias was strongest in cells of the basal membrane layer. With increasing differentiation towards cells of the suprabasal layers, expression of both molecules decreased (Figs. 1B and 2B). CD44s and CD44v6 staining intensity was strong in every one of the leukoplakias tested (n = 22) (Table 1). The percentages of cells that expressed CD44s and CD44v6 were between 50–100% for both proteins (Mean and SEM-CD44s: 93%±2.38%; CD44v6: 81%±3.94%). The mean scores with SEM for CD44s and CD44v6 in oral leukoplakia were 278.9±7.16 and 242±11.7, respectively. The increase in CD44s and CD44v6 expression scores in leukoplakia as compared with normal mucosa was considered significant (Fig. 3) (p-value = 0.05 and 0.03, respectively). As mirrored by the increased percentages of staining, expression of both proteins covered more suprabasal cell layers, as compared to normal mucosa.

### CD44 in head and neck epithelia: squamous cell carcinomas

Next, we analysed the expression of CD44s and CD44v6 in head and neck malignancies. Two carcinomas *in situ* (CIS) stained moderately to strongly with an average and SEM of 100%±0% and 95%±4.95% of cells being positive (Table 1) and yielding scores with SEM of 250±49.9 and 212.5±12.5 for CD44s and CD44v6 (Fig. 3). Hence, scores of CIS were comparable to those of normal mucosa and differences across the sample cohort considered not significant. In head and neck carcinomas, which have been classified by two pathologists as moderately differentiated (G2), all cells (100%) except for two sample (80% and 90%) expressed CD44s and CD44v6 to high levels (+++).Only one sample showed a moderate to strong expression (++/+++) of CD44v6 (Figs. 1D and 2D; Table 1). Average percentages with SEM were 97%±1.9 for CD44s and for CD44v6. Mean scores with SEM for CD44s and CD44v6 were 291.8±5.88 and 287.3±6.88 and both were significantly higher than healthy mucosa (Fig. 3).

For the case of carcinomas diagnosed as poorly differentiated (G3) staining of CD44s and CD44v6 was characterised by a higher inter-tumour heterogeneity. In other words, percentages of positive cells varied from 60%–100% for both proteins and expression levels ranged from negative (−), weak (+), and moderate (++) to strong (+++) (Figs. 1E and 2E; Table 1). These differences as compared to G2 graded carcinomas were also reflected by significantly lower scores along with higher SEMs (208.8±16.68 and 168.8±20.6, respectively). Figure 3 shows additional changes in expression scores that were considered significant or highly significant.

### CD44 in lymphoid cells

**Figure 4:**
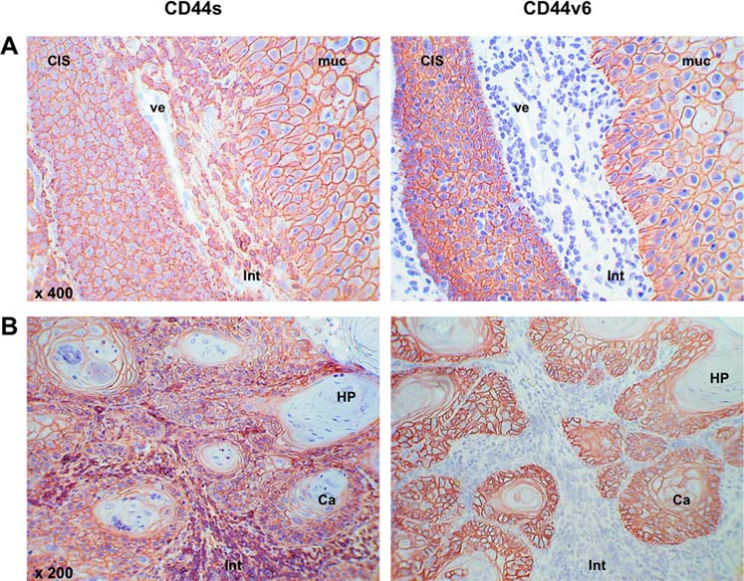
Comparative expression of CD44s and CD44v6 in head and neck epithelia. CD44s and CD44v6 (reddish brown) were visualised in consecutive serial sections of a carcinoma *in situ* (A) and one carcinoma (B). Cell nuclei are stained with hematoxylin (blue). *CIS*: carcinoma *in situ*; *ve*: vessel; *muc*: mucosa; *int*: interstitium; *ca*: carcinoma; *HP*: horn-pearl forming keratinised tumour cells. Shown are representative sections.

**Figure 5:**
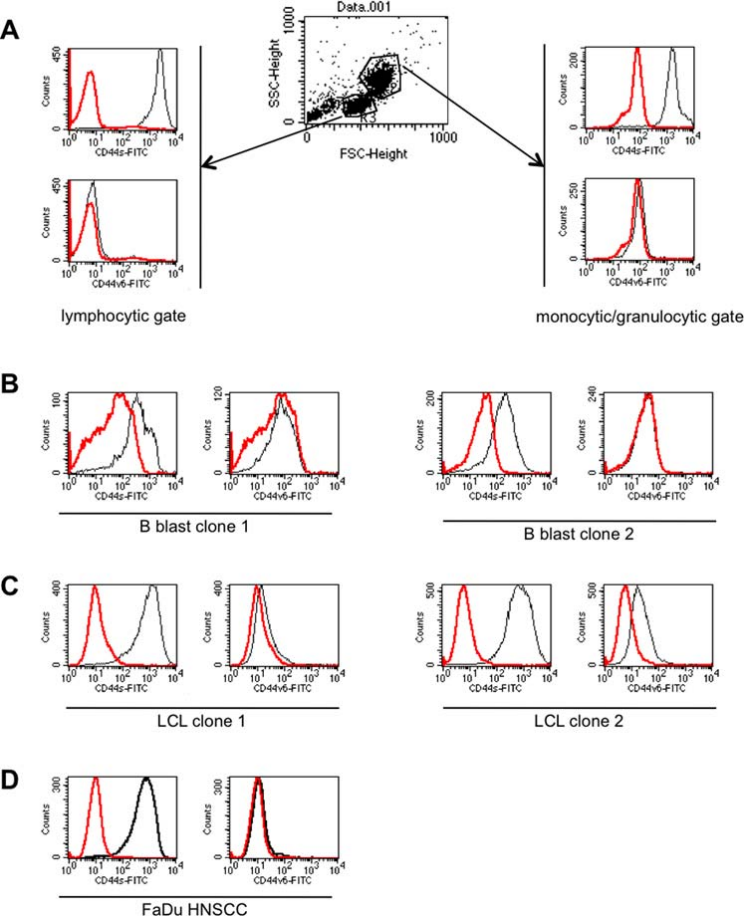
Comparative expression of CD44s and CD44v6 in lymphoid cells. Cell surface expression of CD44s and CD44v6 were assessed on peripheral blood mononuclear cells (A), B blast clones (B), lymphoblastoid cell clones (C), and HNSCC FaDu cells (D) using specific antibodies and flow cytometry. Red line: control; black line: specific antibody. LCL: lymphoblastoid cell lines. Shown are representative results of two independent measurements.

CD44s has been initially described in lymphocytes while CD44v6 was described as being expressed more specifically in epithelia and, more precisely, in tumours thereof [3]. It could already be seen in Figures 1 and 4 that CD44s was not solely expressed in epithelia in head and neck tumour specimens. Rather was CD44s also strongly present at the plasma membrane of lymphocytic cells and was loosely stained in the stroma, too (Figs. 1 and 4, and data not shown). Much in contrast to this, CD44v6 was exclusively expressed on epithelial cells although massive lymphoid infiltrates could be detected (Figs. 2 and 4). For this reason, we compared CD44s and CD44v6 staining directly in consecutive serial sections of the same samples. In one case of CIS, microscopically healthy mucosa was in close proximity of CIS cells and separated by interstitial lymphocytes. This specific case was chosen for comparison of CD44s and CD44v6 expression. CD44v6 was strictly detected in epithelial cells in the normal mucosa and in CIS cells (Fig. 4A right panel). Lymphocytic cells lining vessels and the CIS were devoid of CD44v6. In sharp contrast, CD44s was present in CIS and normal epithelium, but likewise in infiltrating lymphoid cells (Fig. 4A, left panel). The same situation was encountered in HNSCC, where CD44v6 discriminated between carcinoma cells (positive), stroma and lymphocytic cells (negative) (Fig. 4B, right panel). Oppositely, CD44s was present in carcinoma cells and in infiltrating cells to even higher levels (Fig. 4B, left panel). Only exceptions were areas of horn-pearl forming and keratinising tumour cells, which hardly stained for CD44s or CD44v6 (Fig. 4B). These findings were further substantiated upon staining of peripheral blood mononuclear cells. CD44s but not CD44v6 was expressed on cells of the lymphocytic gate and the monocytic/granulocytic gate (Fig. 5A). B blasts, which are human B cells that have been conditionally immortalised upon permanent CD40 ligation [23], expressed CD44s to substantial amounts but entirely lacked CD44v6 (Fig. 5B). Interestingly, Epstein-Barr virus transformed lymphoblastoid cell lines (LCLs) expressed large amounts of CD44s and to varying extent also CD44v6 (Fig. 5C). HNSCC FaDu cells also strongly expressed CD44s, as was described elsewhere [14], and lacked CD44v6 Fig. 5D.

## Discussion

It was roughly a decade ago when scientists revisited the view of tumour origination for the case of leukemia. Coming from a rather *stochastic model*, where every single cell of a tumour would be equipped with tumourigenic potential, scientists moved towards a more *hierarchical model*, with only a subset of cells within malignant tissue that are able to generate tumours. In this model so-called cancer stem cells, CSCs, are the founders of tumours. CSCs display to some degree properties of normal stem cells including self-renewal, pluripotency, and limitless proliferation [24]. The remaining cancer cells that lack these properties are characterised by a restricted or even no growth capacity at all *in vivo*. Data supporting the notion of CSCs in solid tumours emerged in early 2000, when Al-Hajj *et al.* characterised breast cancer-initiating cells [12]. Breast CSCs displayed a CD44^+^/CD24^−^/lin^−^/ESA-1^high^ cell surface phenotype. Amazingly, as few as 100 cells sufficed to recapitulate human carcinogenesis *in vivo* upon inoculation in immunodeficient murine recipients. Importantly, resulting tumours were of mixed phenotype, included all epithelial cells seen in the primary tumours, and could be serially transplanted. The interacting protein CD44 and EpCAM [5] were part of the CSC signature in not only breast cancer but also colon [13]. With respect to EpCAM (ESA-1) [25], [26], it was consistently the phenotype of high-level expression that characterised CSCs. This was markedly different for CD44, whose simple expression discriminated CSCs from non-tumourigeneic cells in head and neck malignancies [14]–[16].

In the present study the expression of the standard form CD44s and the tumour-associated alternative splice variant CD44v6 were assessed in epithelia of the head and neck, including pre-malignancies and full-blown carcinomas. Surprisingly and somewhat in contradiction to available data [18]–[20], CD44s was abundantly expressed in most tumour cells within HNSCC. Percentages of CD44s-positive cells in normal head and neck epithelial cells ranged from 60% to 95% with strong staining intensities. In moderately differentiated HNSCC, CD44s was present in 80–100% of tumour cells and additionally in virtually all infiltrating lymphocytes. Prince *et al.* reported on thirty-three patients suffering from diverse HNSCC including tongue, larynx, floor of the mouth, oropharynx, and maxillary sinus [15], [16]. In these cases CD44s percentages within tumours ranged from 0.1% to 41.72%, hence low levels, and were typically below 10% of the total tumour cell population, which remained unseen in our specimens. It is important to note that the same antibody specific for CD44s was used in both studies and that antibody concentrations used herein were down to 100-fold lower. Kawano *et al.* on the other hand described that 75.4% of samples of patients suffering from mesopharyngeal cancers (n = 57) were positive for CD44s and 78.9% were positive for CD44v6 [27]. These dimensions fit the percentages of CD44s and CD44v6 expressing cells assessed herein.

The alternative splice variant CD44v6 showed an expression pattern similar to CD44s in epithelial areas, independently of the transformation status. We could only detect an over-expression of CD44v6 in grade 2 carcinomas as compared to normal epithelium. However, in contrast to CD44s, CD44v6 was not expressed in infiltrating lymphocytic cells but remained restricted to epithelia. CD44v6 was of interest owing to its potential to increase proliferation via the MAP kinase and Ras signalling pathways [28], [29]. Also, CD44v6 expression was correlated with a reduced disease-free survival in laryngeal malignancies [30]. We could however not confirm a clear association of CD44v6 with malignancy for HNSCC.

In summary, we show that CD44s is abundantly expressed in head and neck carcinomas. This is in sharp contrast to previous reports on CD44s and cancer stem cells in HNSCC, and may rely on different technologies used to visualise CD44s-positive cells, *i.e.* flow cytometry *versus* immunohistochemistry. Similar inconsistencies have been reported recently concerning CD133, a marker for CSCs in numerous entities including colon, brain, prostate, liver, and pancreas [17]. Shmelkov *et al.* demonstrated that CD133 is ubiquitously expressed in murine and human colon carcinoma cells [17]. Further on cancer stem cell potential was not restricted to CD133-positive cells. Both, CD133^+^ and CD133^−^ cells bore long-term tumourigenic potential in immunodeficient mice. Though the principle and existence of CSCs remains undoubted, the actual nature of markers of tumour-initiating cells, their usage, expression, and signification need more attention.

## Materials and Methods

### Tissue samples

Samples of normal mucosa (n = 10), oral leukoplakia (n = 22), carcinomas *in situ* (CIS) (n = 2), moderately differentiated carcinomas (G2; n = 11), and poorly differentiated carcinomas (G3; n = 13) were obtained after written informed consent during routine surgery according to institutional ethics approval by the ethics committee of the local medical faculty (*Ethikkommision der Medizinischen Fakultät der Ludwig-Maximilians-Universität München*). All samples were embedded in tissue-tek (Sakura, Fintek, NL) and snap-frozen in liquid nitrogen before processing to 4μm thick consecutive cryosections. All specimens had been examined and confirmed by two pathologists during routine clinical diagnosis and histopathologic examination after hematoxylin/eosin staining (see Figure S2 for examples of H/E staining).

### Immunohistochemistry

The mouse anti-human CD44s (mouse IgG2b, lot # 86572, BD Pharmingen, Heidelberg, Germany) and anti-human CD44v6 (mouse IgG1, lot # 212115, Novocastra, New Castle upon Tyne, UK) primary antibodies were used. Immunostaining was performed using the avidin-biotin-peroxidase complex method (Vectastain, Vector laboratories, Burlingame, CA, USA) according to the manufacturerś protocol. Briefly, after fixation in acetone (10 min), endogenous peroxidase activity was inhibited upon treatment with 0.03% H_2_O_2_/PBS (10 min). Before specific staining, unspecific antigenic sites were blocked with normal horse serum. Sections were then incubated with the respective primary antibody for 1 hour at room temperature (RT) followed by incubation with biotinylated anti-mouse immunoglobulins and then with avidin-biotin-peroxidase complex (30 minutes at RT for each step). After each step, sections were washed with PBS. Specific peroxidase activity was visualized with 0.05% 3-amino-9-ethylcarbazol (Sigma, Deisenhofen, Germany) and 0.02% H_2_O_2_/0,1M Na-acetat buffer pH5.5 as a substrate. Counterstaining was performed with Mayers hematoxylin and eosin. As a negative control, staining was performed with mouse pre-immune serum instead of specific antibodies. Assessment of percentages of positive cells was performed across the entire sample in a blinded fashion upon light microscopy by two investigators and is given as the mean of both percentages assessed in steps of 5%.Percentages of staining refer to epithelial/tumour cells within samples.

### Flow cytometry

Peripheral blood mononuclear cells, B blasts, lymphoblastoid cell lines, or head and neck squamous cell carcinoma cells FaDu were stained with CD44s- or CD44v6-specific antibodies in combination with a FITC-conjugated anti-mouse secondary antibody. Fluorescence intensities were assessed after washing in PBS in a FACScalibur device (Becton Dickinson, Heidelberg, Germany).

## Supporting Information

Figure S1 Hematoxylin/eosin staining was performed on kryosection of normal epithelium, carcinoma in situ, grades 2 and 3 carcinomas. Stained samples were examined by two pathologists and different areas within specimens were denoted. Shown are representative examples of the entire cohort used in the present study.
(4.16 MB TIF)

Figure S2 Negative controls for immunohistochemistry. Normal mucosa and grade 2 carcinoma were stained with murine pre-immune serum in combination with standardised detection systems. Nuclei were counter-stained with hematoxylin. Shown are representative results from the cohort analysed in the present study.
(1.57 MB TIF)
