# Supplementary figures and images for: CD44s and CD44v6 Expression in Head and Neck Epithelia
Source: PLoS One. 2008 Oct 9;3(10):e3360. doi: 10.1371/journal.pone.0003360 (PMC2566597; doi:10.1371/journal.pone.0003360)

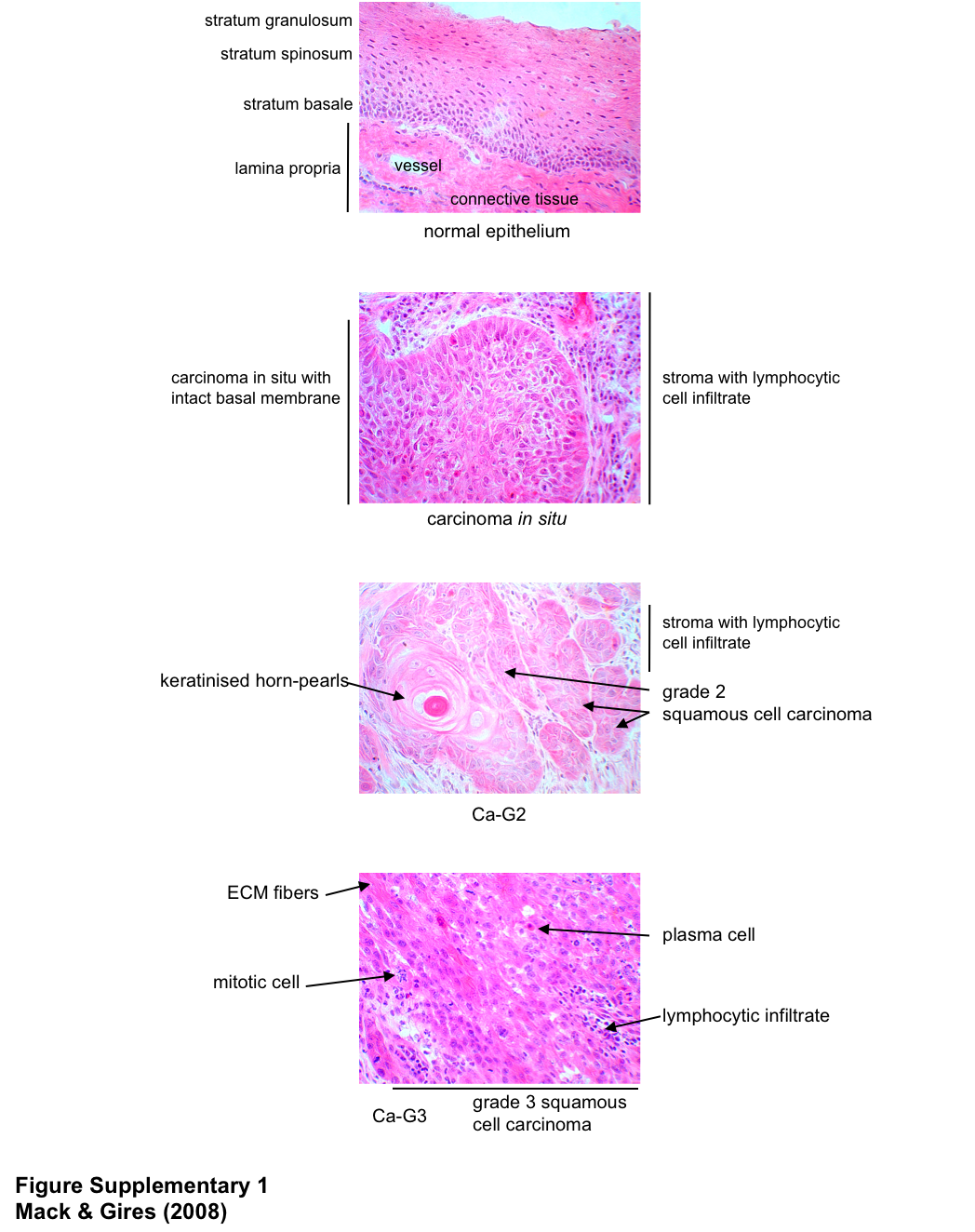

Supplement: Figure S1 — Hematoxylin/eosin staining was performed on kryosection of normal epithelium, carcinoma in situ, grades 2 and 3 carcinomas. Stained samples were examined by two pathologists and different areas within specimens were denoted. Shown are representative examples of the entire cohort used in the present study. (4.16 MB TIF) [file pone.0003360.s001.tif]

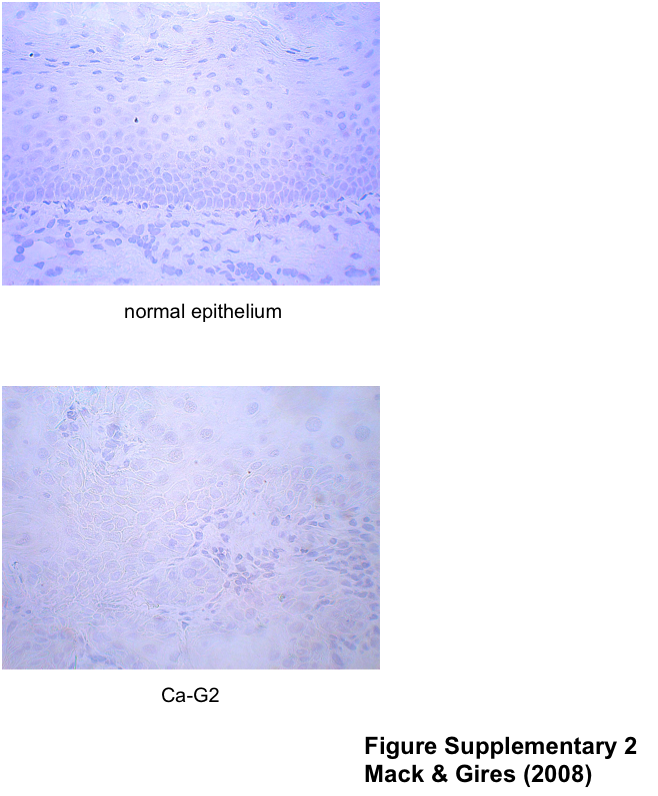

Supplement: Figure S2 — Negative controls for immunohistochemistry. Normal mucosa and grade 2 carcinoma were stained with murine pre-immune serum in combination with standardised detection systems. Nuclei were counter-stained with hematoxylin. Shown are representative results from the cohort analysed in the present study. (1.57 MB TIF) [file pone.0003360.s002.tif]
